# Logarithm conformal mapping brings the cloaking effect

**DOI:** 10.1038/srep06862

**Published:** 2014-10-31

**Authors:** Lin Xu, Huanyang Chen

**Affiliations:** 1 College of Physics, Optoelectronics and Energy, Soochow University, Suzhou 215006, the People's Republic of China

## Abstract

Over the past years, invisibility cloaks have been extensively discussed since transformation optics emerges. Generally, the electromagnetic parameters of invisibility cloaks are complicated tensors, yet difficult to realize. As a special method of transformation optics, conformal mapping helps us design invisibility cloak with isotropic materials of a refractive index distribution. However, for all proposed isotropic cloaks, the refractive index range is at such a breadth that challenges current experimental fabrication. In this work, we propose two new kinds of logarithm conformal mappings for invisible device designs. For one of the mappings, the refractive index distribution of conformal cloak varies from 0 to 9.839, which is more feasible for future implementation. Numerical simulations by using finite element method are performed to confirm the theoretical analysis.

Transformation optics (TO) has been a powerful tool to design versatile devices to manipulate the electromagnetic field, started from two pieces of work on invisibility cloak^1^,^2^. TO's fundamentality is that there is a form invariance under coordinate transformations in Maxwell's equations. As a special method of TO, conformal mapping^1^ gives us a simpler way to control light rays/waves in two dimensions by using isotropic materials. Meanwhile, quasi-conformal mappings, another numerical method has also been applied to design isotropic devices such as carpet cloaks^3^,^4^,^5^,^6^,^7^,^8^,^9^,^10^,^11^,^12^. Numerous designs have been proposed based on different kinds of conformal mappings, such as invisibility cloak based on Zhukowski conformal mapping^1^,^13^,^14^, conformal lenses based on power conformal mapping^15^, wave bend device based on logarithm conformal mapping^16^, directional emitter based on Möbius conformal mapping^17^, and unidirectional radiation devices based on logarithm conformal mapping with a linear term^18^. As far as we know, only two of these devices have been implemented^19^,^20^ to date. What renders experimental fabrication challenging? Among a clutch of hindrances, it is the board range of the refractive index distribution. For example, in the cloaking design, refractive index in the first conformal cloak^1^ ranges from 0 to 36. Recently, the maximum value has been dramatically reduced to 13 by Wu et al^14^. However, it is still difficult to realize in practice. Given that, it is critical to further reduce the maximum value.

In this paper, we propose two new kinds of logarithm conformal mappings for cloaking design, inspired by the mapping used in Ref. 18. We find several interesting properties in the logarithm conformal mappings after introducing two dual logarithm terms with one linear term. We plot virtual space and physical space of both mappings to present their geometry. With two kissing mirrored Maxwell's fish-eye lenses applied in the second Riemann sheet of the virtual space, we can design a conformal cloak with a refractive index profile ranging from 0 to 9.839, which eases experimental implementation. All the numerical simulation results are obtained by the commercial FEM software COMSOL.

## Results

To begin with, let's briefly review TO by using conformal mapping^1^,^13^, which relates to complex analytic functions widely employed in two dimensional problems. It maps virtual space to physical space, and vice versa. Here we regard *w* complex plane (*w* = *u* + *vi*) as virtual space and *z* complex plane (*z* = *x* + *yi*) as physical space. We assume virtual space with a refractive index distribution *n*'(*u*,*v*) and physical space with a refractive index distribution *n*(*x*,*y*). The governing equation of amplitudes *ψ* of the two polarizations of light in physical space is Helmholtz equation^1^, 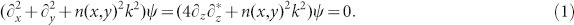
where *ψ* denotes the electric-field component, and *k* is wave vector. Under a conformal mapping *w* = *w*(*z*), Helmholtz equation in virtual space changes into, 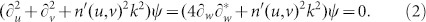
The relationship of refractive index distribution in virtual space and that in physical space is written as, 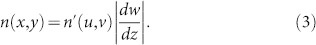
As Helmholtz equation can be derived from Maxwell's equations, the physics law of Helmholtz equation is invariant in virtual space and physical space under conformal mapping^21^. In this paper, we only consider transverse electric (TE) polarized wave for simplicity and assume that the permeability is 1.

Now, we introduce two new kinds of logarithm conformal mappings. The first is, 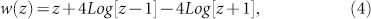
while the second is, 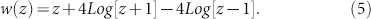
In the above two mappings, dual logarithm terms (*Log*[*z* + 1] and *Log*[*z* − 1]) and one linear term (*z*) are included. The coefficients of dual logarithm terms are both 4. The positions of the singularities ((1+0*i) and (-1+0*i)) are symmetric to the origin point. The relationship between virtual space and physical space of the two new logarithm conformal mappings is shown as follows.

**Figure 1:**
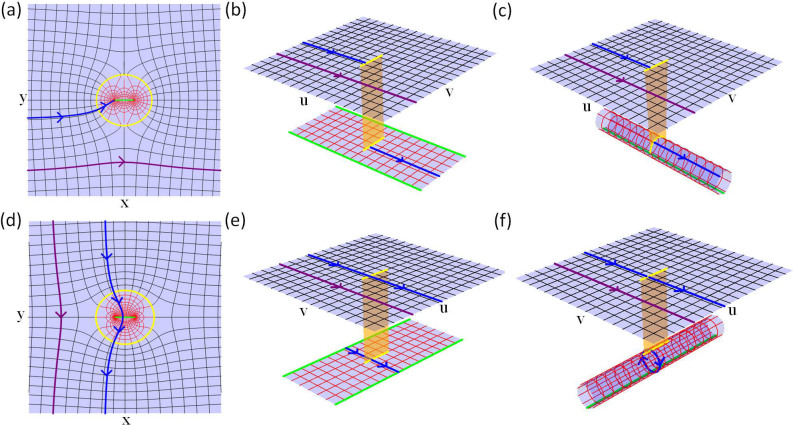
Virtual space and physical space for two new kinds of logarithm conformal mappings. (a) Physical space of the first mapping. (b) Virtual space of the first mapping. (c) The equivalent diagram of virtual space of the first mapping. (d) Physical space of the second mapping. (e) Virtual space of the second mapping. (f) The equivalent diagram of virtual space of the second mapping.

In the first mapping written as Eq. (4), the contours of real part and imaginary part on *z* plane are shown in Fig. 1(a) (physical space), while the contours of *u* and *v* on *w* plane are plotted in Fig. 1(b) (virtual space). Virtual space (Fig. 1(b)) consists of two Riemann sheets, with the upper sheet an infinite complex plane (meshed with black lines) and the lower sheet a finite complex plane (meshed with red lines). The width of the lower sheet in *v* direction is 8*π* in Fig. 1(b). These two sheets are connected with a branch cut (the yellow line) along *v* direction. In Fig. 1(b), the length of the yellow line branch cut is *l*_1_ = 11.07394. No boundary line is drawn in the upper sheet to represent its infinity both in *u* and *v* direction, while two green boundary lines are sketched in the lower sheet to denote its finitude in *v* direction but infinity in *u* direction. Because of this conformal mapping, one point at the yellow line branch cut in virtual space is mapped to two points at a yellow circle-like closed curve in physical space (see in Fig. 1(a)). Therefore, the yellow line branch cut blows up in *u* direction to become the closed curve. The upper Riemann sheet in virtual space is mapped to the region outside the closed curve in physical space, while the lower one in virtual space is mapped to the region inside the closed curve in physical space. After understanding the relationship between virtual space and physical space of the first conformal mapping, let us see how light rays travel in both spaces. One light ray (blue line with arrows) in Fig. 1(b) will enter the lower sheet to the infinity if it impinges the branch cut. Corresponding to physical space in Fig. 1(a), the light ray (blue curve with arrows) will enter one of the singularities once it impinges the closed curve. As for another light ray (purple line with arrows) in Fig. 1(b), it will go ahead without touching the branch cut. Corresponding to physical space in Fig. 1(a), the light ray (purple curve with arrows) continues its way outside the closed curve. It is noted that if light ray enters the lower sheet obliquely (i.e., not parallel to the green boundaries), it will impinge the boundaries. Due to the logarithm mapping, the finite space in the lower sheet is a periodical function in *v* direction. Therefore, after impinging one of the boundaries, the ray will appear from another boundary without changing its propagation direction, see Fig. S1 (b) in the Supplementary Information. We can actually re-plot the virtual space of Fig. 1(b) with an equivalent diagram in Fig. 1(c). The cylindrical surface represents the lower sheet. The perimeter of the cylindrical surface equals to the width of the lower sheet in *v* direction. The two green boundary lines in Fig. 1(b) are now corresponding to the green generatrix in Fig. 1(c). If light ray enters the lower sheet parallel to the green generatrix, it will continue to propagate in a straight line at the cylindrical surface, as shown by the blue line with an arrow in Fig. 1(c). However, if light ray enters the lower sheet obliquely, it will propagate in a helix curve at the cylindrical surface, as shown in Fig. S1(c). In physical space, it will enter one of the singularities in a spiral curve, see in Fig. S1(a).

In the second mapping as Eq. (5), similar to the first one, the contours of real part and imaginary part on *z* plane are shown in Fig. 1(d) (physical space), while the contours of *u* and *v* on *w* plane are plotted in Fig. 1(e) (virtual space). Virtual space (Fig. 1(e)) consists of two Riemann sheets, with the upper one also an infinite complex plane (meshed with black lines) and the lower sheet a finite complex plane (meshed with red lines). The width of *v* direction is 8*π* in Fig. 1(e). The two sheets are connected with a branch cut (a yellow line) along *u* direction, different from that in the first case. In Fig. 1(e), the length of the yellow line branch cut in virtual space is *l*_2_ = 11.54518. Because of this conformal mapping, one point at the yellow line branch cut in virtual space is mapped to two points at the yellow circle-like closed curve in physical space. Again, the yellow line branch cut blows up in *v* direction to become a closed curve. The upper Riemann sheet in virtual space is mapped to the region outside the closed curve in physical space, while the lower one in virtual space is mapped to the region inside the closed curve in physical space. Similar to the first mapping, we can also re-plot the virtual space of Fig. 1(e) with an equivalent diagram in Fig. 1(f). After understanding virtual space and physical space of the second conformal mapping, we can derive the trajectory of light rays in both spaces. If a light ray (blue line with arrows) normally enters the lower sheet through the branch cut, it will impinge one of the boundary of the lower sheet and appear at another boundary, as shown in Fig. 1(e). After that it will impinge the branch cut again at the same point and come back to the upper sheet. If we describe this propagation in the equivalent diagram, the light ray will become a circle at the cylindrical surface, as shown in Fig. 1(f). Corresponding to Fig. 1(d) in physical space, it (blue curve with arrows) will pass through the closed curve twice. As for another light ray (purple line with arrows) in Fig. 1(d), it will go ahead without touching the branch cut. Corresponding to Fig. 1(f) in physical space, it (purple curve with arrows) continues its way outside the closed curve. If light ray enters the lower sheet obliquely, similar to the first mapping, it will impinge one of the boundaries of the lower sheet, and appear at another boundary, as shown in Fig. S1(e). Sometimes, it will come back to the branch cut at a different point and enter the upper sheet again. Or, it will not come back but continue to propagate in the lower sheet. In the equivalent diagram, the propagation is in helix curves, as shown in Fig. S1(f). In physical space, sometimes the light will impinge the closed curve twice and continue to propagate. Or, it will enter one of the singularities in a spiral curve, see in Fig. S1(d).

In these two conformal mappings, the second sheet is finite in one direction because of the periodical property of the logarithm terms, which is quite different from the Zhukowski conformal mapping mostly used for cloaking design^1^,^13^,^14^. We notice that both mappings allow us to put two kissing mirrored Maxwell's fish-eye lenses (proposed by Wu et al^14^) in the second sheet, leading light rays to return to the upper sheet with their positions and directions conserved. The refractive index profile of a mirrored Maxwell's fish-eye lens with a radius *r*_0_ is written as, 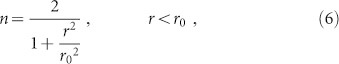
which helps us make a perfect imaging^22^. The refractive index of a mirrored Maxwell's fish-eye lens ranges from 1 at the outer boundary to 2 at the center. It is worth mentioning that many kinds of refractive index profile^1^,^13^,^23^ could be used in the second sheet for designing invisibility cloaks. The reason why we choose two kissing mirrored Maxwell's fish-eye lenses mentioned in Ref. 14, 24 is that, (1) the cloaked region is bounded with a perfect electric conductor (PEC), no matter what in the cloaked region will not affect the cloaking functionality; (2) the whole device is of a mirror symmetric property; (3) it has more of a refractive index range feasible for implementation. The maximum value of the refractive index profile for conformal cloaks could be further reduced as shown in the following.

**Figure 2:**
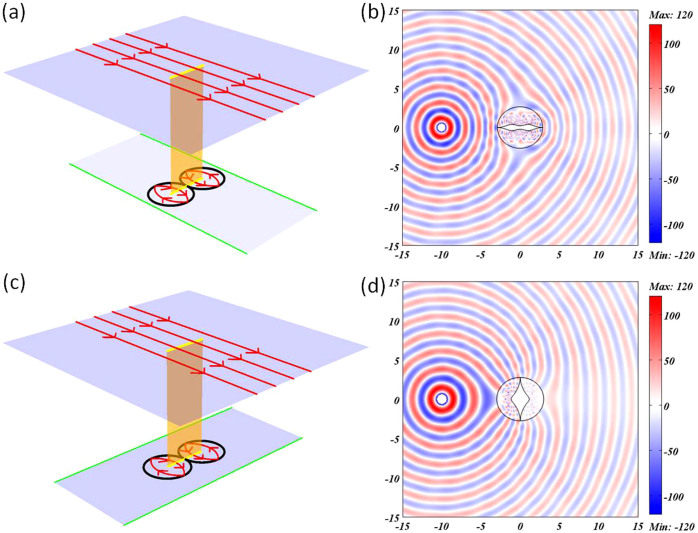
The geometrical light rays in virtual space and the wave cloaking effect in physical space. (a) The light rays in virtual space of the first conformal mapping, the two kissing black circles are PEC boundaries of two kissing mirrored Maxwell's fish-eye lenses. (b) The electric field pattern for the conformal cloak of the first mapping. In the numerical simulation, the point source is placed at the coordinate (-10,0) and its frequency is 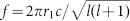 with *l* = 16. (c) The light rays in virtual space of the second conformal mapping, the two kissing black circles are PEC boundaries of two kissing mirrored Maxwell's fish-eye lenses. (d) The electric field pattern for the conformal cloak of the second mapping. In the numerical simulation, the point source is placed at the coordinate (-10,0) and its frequency is 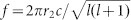 with *l* = 16.

**Figure 3:**
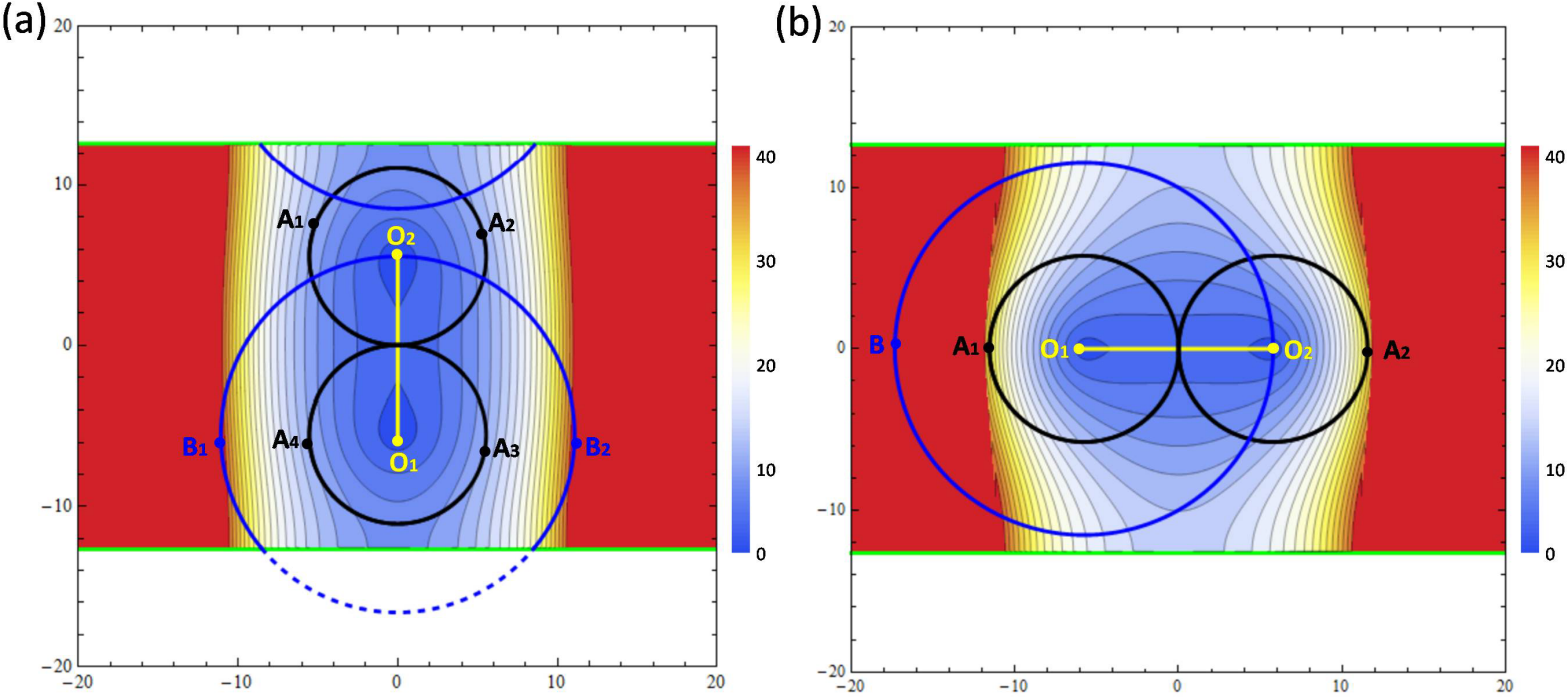
The contour plot of |*dw*/*dz*| in the lower sheet of virtual space. (a) The contour plot of |*dw*/*dz*| for the first conformal mapping. (b) The contour plot of |*dw*/*dz*| for the second conformal mapping.

Let us now look at the first conformal mapping. The width of the second Riemann sheet is 8*π*, slightly bigger than twice of the length of the branch cut in virtual space. Therefore the radii of the mirrored Maxwell's fish-eye lenses are set as *r*_1_ = *l*_1_/2 = 5.53697. As shown in Fig. 2(a), if we put two kissing lenses mentioned above in the lower Riemann sheet in virtual space, all rays impinging the branch cut will enter the lower sheet, propagate in closed circular arc trajectories and return to the upper sheet after reflecting twice at the PEC boundaries of Maxwell's fish-eye lenses. In physical space, all rays will propagate around the PEC boundary (the boundary of the white region in Fig. 2(b)) and leave the device as if nothing is there. According to Eqs. (3), (4) and (6), the refractive index distribution from the first conformal mapping is, 
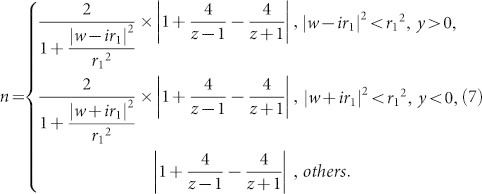
The detailed calculation can be found in Ref. 1, 14, 15. Before we move on, let us examine schematically why we use two fish-eye lenses instead of one. In Fig. 3(a), the ribbon pattern is the lower sheet in virtual space of the first conformal mapping in Fig. 2(a). The yellow line with two endpoints (O_1_ and O_2_) is the branch cut. Two green lines are the boundaries of the lower sheet, which could be glued together if we roll up the sheet into a cylindrical surface. We plot the contour of |*dw*/*dz*| in the lower sheet, which is small near the branch cut, and gradually grow into infinity away from the branch cut. We use uniform red color to represent large |*dw*/*dz*| in regions far from the branch cut. By applying two kissing mirrored Maxwell's fish-eye lenses (two black circles), whose centers are the endpoints of the branch cut (radii of each lens is half length of the branch cut), as the refractive index of the lenses ranges from 1 to 2, the maximum value of the refractive index of the whole cloaking device shows up around points of A_1_, A_2_, A_3_ and A_4_, which are near the contour line of 9. These points are symmetric to the branch cut. The reason why we set the radii of the lenses half length of the branch cut is that it constrains the region of two lenses to have a lower upper bound of refractive index. However, if we put one Maxwell's fish-eye lens (shown in blue curves) whose center is O_1_, the maximum value shows up around points of B_1_ and B_2_, which are near the contour line of 40. In this case, the radius of the lens is the length of the branch cut. The blue dashed arc outside the sheet should be mapped to the blue solid arc to form a whole Maxwell's fish-eye lens. Therefore, it is obvious that two fish-eye lenses are better than one in cloaking designs.

As it is known, conformal cloaks can not only work in geometry optics^1^, but also in wave realm when the frequency satisfies the condition^13^,^14^,^24^,^25^

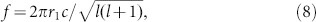
where *l* is an integer and c is the speed of light in vacuum. In Fig. 2(b), we plot the electric field pattern of the cloak for a TE polarized cylindrical wave coming from a point source placed at the position of (-10,0). Good cloaking effect is observed in wave optics at one of the eigen-frequencies (here *l* = 16). The invisibility effect at discrete frequencies is not theoretically rigorous but an approximate expression from numerical simulations and some heuristic analysis from cavity optics^13^,^24^. If the frequency of the incident wave satisfies Eq. (8) in virtual space, eigen-modes of the two kissing mirrored Maxwell's fish-eye lenses in the lower sheet could be excited. There will be a phase delay of an integer time of 2*π* for the incident wave after it leaves the branch cut, leading to the invisibility effect for wave. To visualize the cloaking effect, we plot the electric field patterns of both conformal cloak and a PEC structure for comparison in Fig. S2 in Supplementary Information. We also zoom in the field pattern near the cloaking region to show the details in Fig. S2(b). Without the conformal cloak, the inner PEC region will cause obvious scattering, as in Fig. S2(c).

For the second conformal mapping, the radii of mirrored Maxwell's fish-eye lenses are set as *r*_2_ = *l*_2_/2 = 5.77259. As shown in Fig. 2(c), if we still put two kissing mirrored Maxwell's fish-eye lenses on the second sheet in virtual space, similar to the first case, all rays entering the lower sheet will return to the upper sheet after closed circular arc orbits with reflection for twice at the PEC boundaries. From Eqs. (3), (5) and (6), the refractive index distribution for the second conformal mapping can be written as follows, 
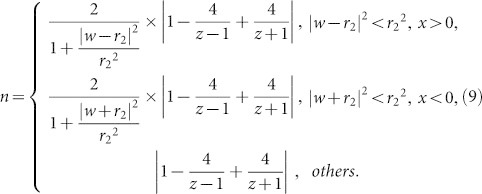
Similar to the first conformal mapping, we also plot the contour of |*dw*/*dz*| in the lower sheet in Fig. 3(b) to illustrate the property of refractive index distribution. If two kissing mirrored Maxwell's fish-eye lenses are applied in the lower sheet with their centers at O_1_ and O_2_ (two black circles), the maximum value of refractive index shows up near the points of A_1_ and A_2_. If only one lens is applied with its center O_1_ (the blue circle), the maximum value shows up near the point of B, which is very large. Therefore from Fig. 3, it is clear that two kissing mirrored Maxwell's fish-eye lenses is better than one lens in achieving a lower refractive index upper bound.

At one of the eigen-frequencies of kissing mirrored Maxwell's fish-eye lenses, good cloaking effect can also be observed, as shown in Fig. 2(d). The point source is placed at the position of (-10, 0) and its frequency is 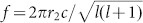 with *l* = 16. The field pattern near the cloaking region is zoomed in Fig. S2(e). Likewise, without the conformal cloak, the PEC region will cause scattering, as shown in Fig. S2(f).

**Figure 4:**
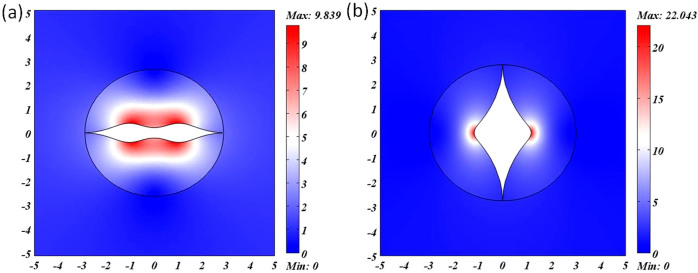
The refractive index distributions of the two designs. (a) The refractive index distribution of Fig. 2 (b). (b) The refractive index distribution of Fig. 2 (d).

So far, we have shown that two kinds of logarithm conformal mappings could be utilized for cloaking design, though there is a little scattering due to the impedance mismatching at the branch cut. Now we plot the whole refractive index distribution in both cases based on Eq. (7) and Eq. (9). The refractive index distribution from the first mapping is shown in Fig. 4(a), varying from 0 to 9.839, while the range of refractive index from the second conformal mapping (shown in Fig. 4(b)) goes from 0 to 22.043. It is clear that the range from the first mapping is closer to future experiments.

Finally, such devices can also be employed for imaging illusion. For example, with them two point sources in phase will appear as one with stronger intensity, or two point sources with anti-phase will be invisible, see in Fig. S3 in the Supplementary Information.

## Discussion

In conclusion, we have proposed two kinds of logarithm conformal mappings for cloaking designs. By applying two kissing mirrored Maxwell's fish-eye lenses on the lower sheet in one of the conformal mappings, the range of refractive index goes from 0 to 9.839, which is more feasible for implementation when compared to the previous work^1^,^14^. In our designs, the symmetric properties of the discussed conformal mappings and those of two kissing mirrored Maxwell's fish-eye lenses lower the refractive index distribution. We can tune the coefficients of duel logarithm terms and the positions of singularities to optimize the refractive index distribution of invisibility cloaks. We believe that the maximum value of refractive index for conformal cloaks could be further reduced if new conformal mappings are proposed to construct virtual space and physical space. Hopefully a real conformal cloaking device could be brought about. In addition, these two conformal mappings can also be good for other designs, perhaps an artificial electromagnetic wormhole^26^.

## Author Contributions

H. C. conceived the idea, and L. X. did the theoretical calculations and the numerical simulations. H. C. and L. X. wrote the manuscript.

## Supplementary Material

Supplementary Information Logarithm conformal mapping brings the cloaking effect
