# Supplementary material for: Logarithm conformal mapping brings the cloaking effect
Source: Sci Rep. 2014 Oct 31;4:6862. doi: 10.1038/srep06862 (PMC4215307; doi:10.1038/srep06862)

Supplementary Information:

Logarithm conformal mapping brings the cloaking effect

Lin Xu, and Huanyang Chen*

College of Physics, Optoelectronics and Energy, Soochow University, Suzhou 215006, the People's Republic of China

*kenyonchy@gmail.com

Figure S1 | Oblique incidence of light rays in physical space and virtual space for the two logarithm conformal mappings. (a) A blue light ray spirally goes to one of the singularities in physical space of the first mapping. (b) Oblique incident light ray (blue straight line with arrows) impinges the yellow branch cut in virtual space of the first mapping. (c) The trajectory of a light ray (blue straight line and helix with arrows) in an equivalent diagram of virtual space of the first mapping. (d) In physical space of the second mapping, a blue light ray enters one of the singularities, while a purple light ray travels around the singularities and leaves them. (e) In virtual space of the second mapping, two light rays impinge the yellow branch cut obliquely at different angles. The blue one has a smaller incident angle. After entering the lower sheet, it will return to the upper sheet. As for the purple one, it will never come back to the upper sheet but go to infinity because of its larger incident angle. (f) The trajectories of two light rays (blue line/helix and purple line/helix with arrows) in an equivalent diagram of virtual space of the second mapping.

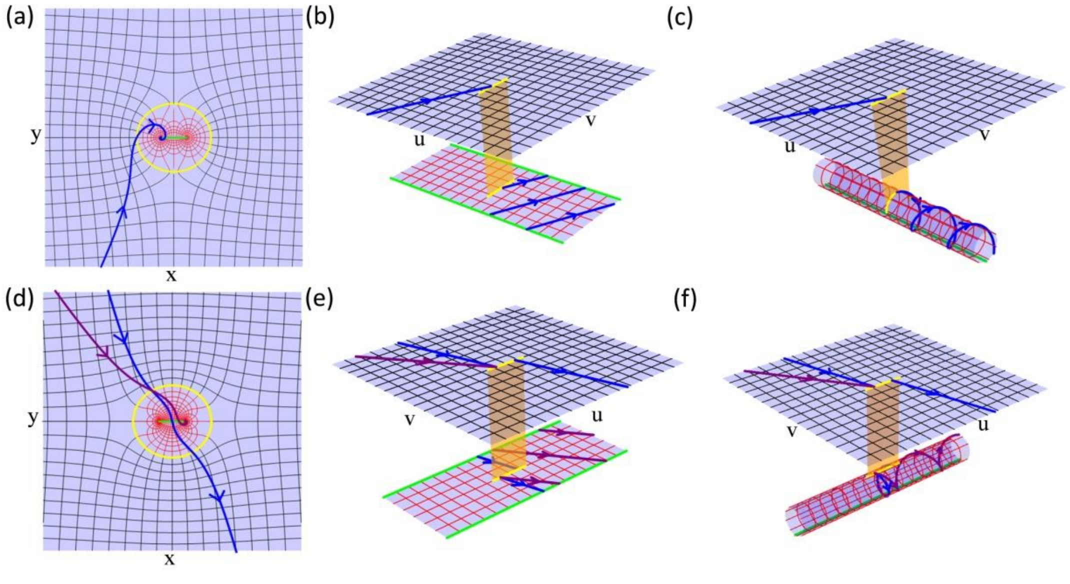

Figure S2 | The comparison of the electric field patterns of our designs and bare PEC structures. (a) The electric field pattern of cloaking design from the first conformal mapping. The cylinder wave front can be recovered behind the cloaking device. (b) The field pattern near the cloaking region of the first design. (c) The electric field pattern of bare PEC. There is scattering behind the PEC for the radiation of point source. In (a), (b) and (c), the point source is placed at the coordinate (-10, 0) and its frequency is $f = 2\pi r_1 \mathrm{c}/\sqrt{l(l+1)}$ with $l = 16$. (d) The electric field pattern of cloaking design from the second conformal mapping. The cylinder wave front can be recovered behind the cloaking device. (e) The field pattern near the cloaking region of the second design. (f) The electric field pattern of bare PEC. There is scattering behind the PEC for the radiation of point source. In (d), (e) and (f), the point source is placed at the coordinate (-10, 0) and its frequency is $f = 2\pi r_2 \mathrm{c}/\sqrt{l(l+1)}$ with $l = 16$.

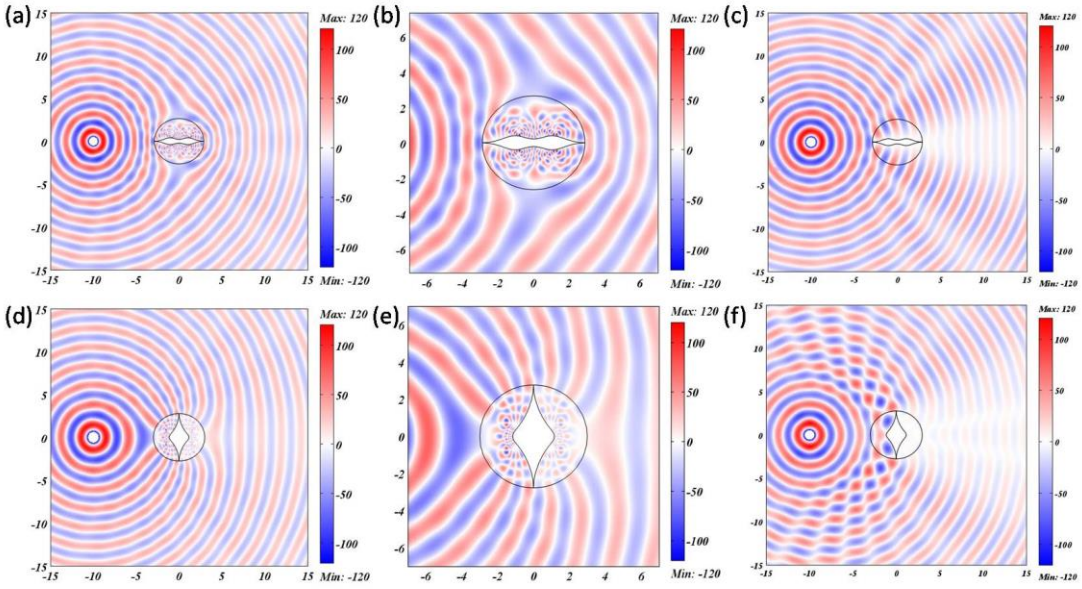

Figure S3 | The imaging properties of two designs. To the first design: (a) Two point sources in phase are placed at (2.1, 1.81985) and (-2.1, 1.81985) respectively. The electric field pattern of two point sources looks like that of one point source with stronger intensity. (b) Two point sources with anti-phase are also placed at (2.1, 1.81985) and (-2.1, 1.81985) respectively. The electric field pattern of two point sources seems disappeared. (c) After ten times scaling down the color bar, the electric field pattern can be revealed, which can be almost ignored in practice. To the second design: (d) Two point sources in phase are placed at (-2.1, 1.98113) and (-2.1, -1.98113) respectively. The electric field pattern of two point sources looks like that of one point source with stronger intensity. (e) Two point source with anti-phase are also placed at (-2.1, 1.98113) and (-2.1, -1.98113) respectively. The electric field pattern of two point sources seems disappeared. (f) After ten times scaling down the color bar, the electric field pattern can be revealed, which can be almost ignored in practice.

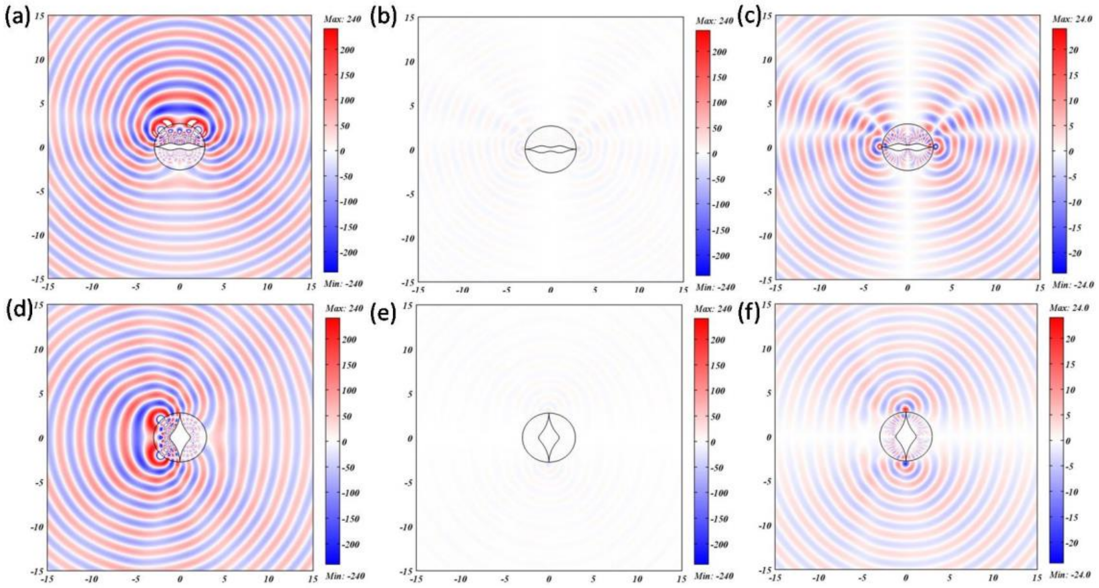

Supplement: Supplementary Information — Logarithm conformal mapping brings the cloaking effect [file srep06862-s1.pdf]
